# mTOR Signaling in Protein Translation Regulation: Implications in Cancer Genesis and Therapeutic Interventions

**DOI:** 10.1155/2014/686984

**Published:** 2014-11-20

**Authors:** Mehvish Showkat, Mushtaq A. Beigh, Khurshid I. Andrabi

**Affiliations:** Department of Biotechnology and Bioinformatics, University of Kashmir, Science Block, Ground Floor, Srinagar, Jammu and Kashmir 190006, India

## Abstract

mTOR is a central nutrient sensor that signals a cell to grow and proliferate. Through distinct protein complexes it regulates different levels of available cellular energy substrates required for cell growth. One of the important functions of the complex is to maintain available amino acid pool by regulating protein translation. Dysregulation of mTOR pathway leads to aberrant protein translation which manifests into various pathological states. Our review focuses on the role mTOR signaling plays in protein translation and its physiological role. It also throws some light on available data that show translation dysregulation as a cause of pathological complexities like cancer and the available drugs that target the pathway for cancer treatment.

## 1. Overview of Translation Initiation

The regulation of translation is crucial for controlling cell growth and proliferation while translation dysregulation results in aberrant growth and tumorigenicity [1]. Translational control is mediated by the 7-methyl-GTP cap structure present at the 5′ termini of all eukaryotic mRNAs where multiprotein complexes are formed during translation initiation. The eukaryotic initiation factor 4G (eIF4G) acts as a scaffold protein for eukaryotic initiation factor 4E (eIF4E) and eukaryotic initiation factor 4A (eIF4A) to form a protein complex eIF4F, which binds to the cap structure and positions the ribosome near the 5′ terminus of mRNA [2]. Because of its low availability, the cap binding protein eIF4E is the rate limiting factor and inhibitory proteins, namely, eIF4E binding proteins (4E-BPs), regulate this process by binding to eIF4E which prevents its association with eIF4G, thus inhibiting protein translation [3]. Upon mitogenic stimulation 4E-BP1 is phosphorylated which is believed to cause its dissociation from eIF4E leading to the subsequent formation of the eIF4F complex, thus resulting in stimulation of translation initiation. Overall translation levels are therefore lowered when 4E-BP1 is active and this activity is thought to be regulated by mTOR dependent phosphorylation [4]. The mTOR activity itself is regulated by growth factors and amino acid availability as well as the energy status of the cell [4]. When mTOR activity is low, 4E-BP1 is hypophosphorylated which allows it to bind efficiently to eIF4E and block translation initiation whereas when mTOR activity is high, 4E-BP1 is phosphorylated causing it to release eIF4E, thus allowing cap dependent translation to begin [5].

## 2. mTOR

TOR is the target of rapamycin, a highly conserved serine/threonine kinase that plays a significant role in controlling cell growth and metabolism [6]. Rapamycin is an antifungal compound produced by the bacteria* Streptomyces hygroscopicus* that was isolated from a soil sample of Rapa Nui islands in the 1970s [7]. It is an anticancer compound that inhibits cell growth and proliferation [8] as well as a potent immunosuppressant that effectively prevents allograft rejection [9]. In 1990s, the isolation of yeast mutants that were resistant to growth inhibition by rapamycin led to the discovery of TOR which was later followed by the identification of the mammalian TOR (mTOR) as the physical target of rapamycin [10].

**Figure 1:**
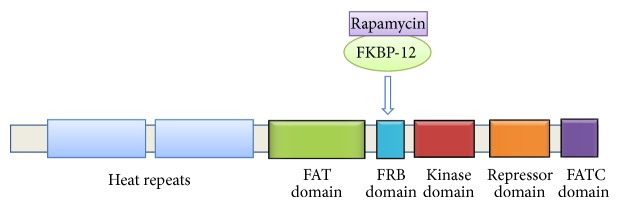
Schematic representation of mTOR domain structure.

mTOR belongs to the phosphatidylinositol 3-kinase (PI3K) kinase-related kinase (PIKK) superfamily as the catalytic domain of PI3K has strong homology with the C-terminus of mTOR [11]. It consists of 2549 amino acids and several conserved domain structures. Tandemly repeated HEAT (for huntingtin, elongation factor 3 (EF3), a subunit of PP 2A, and TOR) motifs comprise its first 1200 amino acids [12]. These tandem HEAT repeats create a superhelical structure with large interfaces that facilitates protein-protein interaction. A FAT (FRAP, ATM, and TRRAP) domain lies downstream the HEAT repeat region which is followed by an FKPB12-rapamycin binding (FRB) domain. Rapamycin binds to FK506 binding protein 12 (FKBP12), thereby inhibiting its enzymatic activity as prolylisomerase, and this rapamycin-FKBP12 complex then binds to the FRB domain of mTOR and inhibits its activity [10]. The FRB domain is followed by a catalytic kinase domain (KD), an autoinhibitory or repressor domain (RD domain), and a FAT carboxy-terminal (FATC) domain (Figure 1). The FATC domain is crucial for the kinase activity of mTOR since deletion of even a single amino acid from this domain inhibits mTOR kinase activity. The FAT domain interacts with the FATC region and this interaction between the two domains might expose the catalytic domain, thus regulating the kinase activity of mTOR [13].

The mTOR pathway regulates cell growth and proliferation in response to mitogen, nutrient, and energy status within the cell and is often dysregulated in various diseases, such as cancer and diabetes [14]. Recent findings have indicated a key role of mTOR signaling in tumorigenesis and activation of the mTOR pathway has been reported in several human cancers [15]. mTOR interacts with different proteins and forms two structurally and functionally distinct multiprotein complexes: mTOR complex 1 (mTORC1) and mTOR complex 2 (mTORC2) [16]. These complexes have important differences in their protein composition, rapamycin sensitivities, upstream signals, and substrates [17].

**Figure 2:**
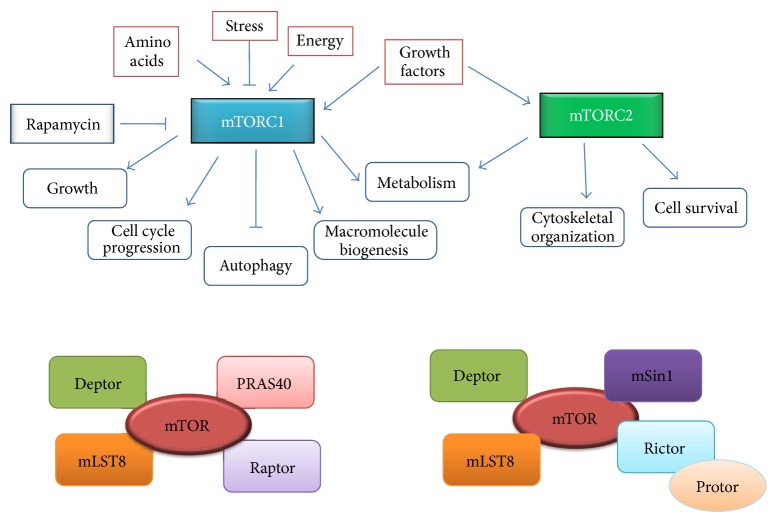
mTORC1 and mTORC2 complexes, different interaction partners, and cellular functions.

*mTORC1.* mTORC1 is composed of mTOR, raptor (regulatory associated protein of mTOR), mLST8 (also called G-protein *β*-subunit-like protein, G*β*L), PRAS40 (proline rich AKT/PKB substrate 40 kDA), and deptor (death domain containing mTOR interacting protein) (Figure 2) [18]. Rapamycin inhibits the mTORC1 activity since rapamycin bound to FKBP12 interacts with either free mTOR or mTOR in the mTORC1 complex causing conformational changes in mTORC1 which are responsible for rapamycin's effect on mTORC1 targets [19]. mTORC1 regulates translation by phosphorylating its various downstream effectors, with S6K1 and 4E-BP1 being the most imperative targets.

Raptor is a 150 kDa mTORC1 binding protein, having a highly conserved N-terminal domain followed by three HEAT repeats and seven WD40 repeats in the C-terminal half [20, 21]. It binds strongly with mTOR via its N-terminal domain containing the HEAT repeats [20] and functions as a vital scaffold protein by linking the mTOR kinase with the mTORC1 substrates 4E-BP1 and S6K1, thus regulating mTORC1 activity in response to various mitogenic signals [22]. The phosphorylation status of raptor governs mTORC1 activity since phosphorylation of raptor on S722/792 by 5′ AMP-activated kinase (AMPK) inhibits mTORC1 whereas mTOR mediated phosphorylation of raptor on S863 is crucial for the activation of mTORC1 in response to mitogen stimulation [23, 24]. PRAS40, another subunit of mTORC1, is a negative regulator of mTORC1 function that was initially identified as an AKT substrate because of its direct phosphorylation at T246 by AKT when activated by insulin [25]. However, later studies showed that PRAS40 inhibits mTORC1 activity and is phosphorylated by mTORC1 on S183 when associated with mTORC1 via raptor and this binding is abolished by mutation of the S183 to aspartate [26, 27]. The role of mLST8 in mTORC1 function is unclear since loss of this protein does not affect mTORC1 activity* in vivo *[28].

mTORC1 activity is regulated by multiple growth factor signals such as insulin and nutrients. mTORC1 is activated by the PI3K/AKT pathway whereas TSC1-TSC2 (tuberous sclerosis complex) inhibits it [14]. Insulin binds to insulin receptor that recruits IRS (insulin receptor substrate) and activates phosphoinositide 3-kinase (PI3K) which induces cell proliferation and cell survival [29]. Besides other proteins, the downstream targets of PI3K include S6K1 and the serine/threonine kinase AKT [30]. Upon activation by growth factors, PI3K phosphorylates the D3 position of phosphatidylinositols producing the second messenger PtdIns(3,4,5)P3 that binds to the pleckstrin homology (PH) domain of AKT and translocates this kinase to the plasma membrane where it gets activated by upstream kinases [31]. PDK1 phosphorylates AKT at T308 and mTORC2 phosphorylates it at S473, both phosphorylations being necessary for the full activation of its kinase activity [32, 33]. Phosphatase and tensin homolog (PTEN) acts as a negative regulator of AKT activation by converting PtdIns(3,4,5)P3 into PtdIns(4,5)P2 which leads to a reduced recruitment of AKT to the cell membrane [34]. The negative regulators of mTORC1, TSC2, and PRAS40 are two of the several downstream substrates of activated AKT that are phosphorylated and inhibited by AKT [35]. Rheb (Ras homolog enriched in brain) GTPase directly binds to the catalytic domain of mTOR and acts as a positive regulator of mTORC1 kinase activity [26]. TSC1 and TSC2 proteins form a complex* in vivo* which negatively regulates mTORC1 by acting as Rheb GAP (GTPase activation protein) that converts Rheb into an inactive GDP bound form [36, 37]. In response to growth factors, AKT directly phosphorylates TSC2 on several distinct residues [35] that prevents the formation of TSC1-TSC2 complex, thus allowing GTPase Rheb to convert back into the GTP-bound active state [38] which leads to mTORC1 activation [39, 40]. Thus PI3K/AKT signalling pathway regulates mTORC1 by phosphorylation and inhibition of TSC2 which impairs its ability to inhibit Rheb, thereby resulting in the subsequent activation of Rheb and mTORC1.

*mTORC2.* mTORC2 is composed of RICTOR (rapamycin-insensitive companion of mTOR), mSin1 (mammalian stress-activated protein kinase (SAPK) interacting protein 1), Protor (protein observed with RICTOR), mLST8, deptor, and PRAS40 [16]. The mTORC2 complex was initially thought to be insensitive to acute rapamycin treatment since this RICTOR containing mTOR complex is not bound by FKBP12-rapamycin [41] but later studies demonstrated that prolonged treatment with rapamycin can inhibit the assembly and function of mTORC2 as well [42]. Deptor and mLST8 are components of both mTOR complexes and whereas deptor negatively regulates mTORC1 as well as mTORC2, mLST8 is essential only for mTORC2 function [28, 43]. mSin1 is another important subunit of mTORC2 that is indispensable for mTORC2 function and integrity because the interaction between RICTOR and mTOR is impaired in its absence [44, 45]. Protor interacts with RICTOR but it is not required for the assembly of other subunits of the mTORC2 complex [46].

The best-characterized function of mTORC2 is the activation of AKT by phosphorylating it on S473 which is essential for the regulation of various important cellular processes such as cell growth, proliferation, glucose metabolism, and apoptosis by activated AKT [33]. The phosphorylation of another conserved motif on AKT is also mediated by mTORC2 [47] and thus mTOR lies both upstream (mTORC2) and downstream (mTORC1) of AKT. The other important substrates of mTORC2 are SGK (serum and glucocorticoid-inducible kinase) and PKC*α* (protein kinase C*α*) [48]. mTORC2 functions also include regulation of PKC maturation and stability [47] in addition to organization of actin cytoskeleton [41].

*Feedback Regulation.* Multiple negative feedback loops regulate the mTOR pathway and various studies have shown that mTORC1 negatively affects the insulin-PI3K-AKT pathway. The insulin receptor substrate 1 (IRS1) is directly phosphorylated by S6K1 at multiple sites which impairs its function and leads to inhibitory effects on the insulin-PI3K-AKT pathway. Growth factor receptor-bound protein 10 (GRB10) is a recently discovered substrate of mTOR that is activated by mTOR phosphorylation and negatively controls insulin-PI3K-AKT signaling pathway by inhibiting the insulin receptor in its active form [49]. The TSC/Rheb axis also regulates mTOR pathway via another feedback loop. Rheb activates mTORC1 but inhibits mTORC2 while TSC1/2 inhibits Rheb/mTORC1 but activates mTORC2 most likely by overcoming the negative feedback loop [50]. TSC2 also interacts with mTORC2 via RICTOR and activates the mTORC2 complex independently of its effects on mTORC1. mTORC2 is inhibited by mTORC1 through a negative feedback loop that involves S6K1, since RICTOR is inhibited by its S6K1 mediated phosphorylation at T1135 [51]. The PI3K-AKT-mTOR pathway is connected with the mitogen activated protein kinase (MAPK) pathway as AKT is activated by treatment with MEK inhibitors [52] and also by another feedback loop since the inhibition of mTORC1 results in the activation of the PI3K pathway as well as the MAPK pathway [53].

## 3. Downstream the mTOR Pathway

The best-characterized downstream effectors of mTORC1 which are phosphorylated by activated mTOR kinase are S6K1 and 4E-BP1.

### 3.1. S6K1

The ribosomal protein S6 kinase (S6K), a serine/threonine kinase that belongs to the AGC kinase family, is an important regulator of cell growth and cell size. The S6K family consists of two genes (S6K1 and S6K2) which share an overall 70% sequence homology [54]. S6Ks have five domains, the N-terminal regulatory domain, catalytic domain, linker domain, an autoinhibitory domain, and the C-terminal domain. S6K1 activity is regulated by sequential phosphorylations at multiple serine/threonine sites. The phosphorylation of four serine/threonine-proline sites in the autoinhibitory domain opens the kinase domain and relieves autoinhibition, which then allows subsequent phosphorylations of critical sites in the kinase domain [55]. mTOR kinase phosphorylates S6K1 at T389 which is the hydrophobic motif (HM) phosphorylation site [56, 57] followed by the PDK1 mediated phosphorylation at T229 present in the activation loop (AL) which leads to full activation of S6K1 [58]. The N-terminus of S6K1 has a conserved TOR signaling (TOS) motif that interacts with raptor and enables mTORC1 mediated phosphorylation of S6K1 [59]. The C-terminal region of S6K1 has an RSPRR motif which is important for the inhibitory role of this region, because a negative regulator of S6K1 binds to this motif [60] and a deletion mutant of S6K1 lacking the C-terminal region is phosphorylated by mTORC2 [61]. Although PDK1 is a rapamycin resistant kinase, T229 is also lost along with T389 upon rapamycin treatment therefore suggesting that T229 phosphorylation is dependent on T389 phosphorylation. On the other hand it has been reported that the T389 phosphorylation is not required for T229 phosphorylation [62]. More recently, we have reported that these phosphorylations might be coordinate instead of being sequential and the loss of these HM and AL phosphorylations is consequential and not because of inhibition of S6K1 by rapamycin [63]. Earlier we had also shown that the activity and rapamycin sensitivity of the exogenously expressed S6K1 are independent of any TOR dependent phosphorylations [64].

The ribosomal protein S6 (rpS6) was the first identified substrate of S6K. It is phosphorylated on five C-terminal serine sites by S6Ks in the following sequential order: S236 > S235 > S240 > S244 > S247 [65]. The study of rpS6P^/−^ knock-in mice (in which alanine residues replaced the rpS6 phosphorylation sites) established that rpS6 phosphorylation is crucial for cell size and proliferation since cells isolated from rpS6P^−/−^ displayed defective cell growth [66, 67]. S6K1 is very essential for regulating cell and body size as S6K1 null mice are much smaller at birth because of a decrease in the size of all organs [66] and a majority of dS6K null Drosophila show embryonic lethality [68]. The study of rpS6P^−/−^ mice has also confirmed that the translation of mRNAs having a 5′ terminus oligopyrimidine tract (5′TOP mRNAs), a process that was earlier considered to be regulated by rpS6 phosphorylation, is not dependent on this event [69].

**Figure 3:**
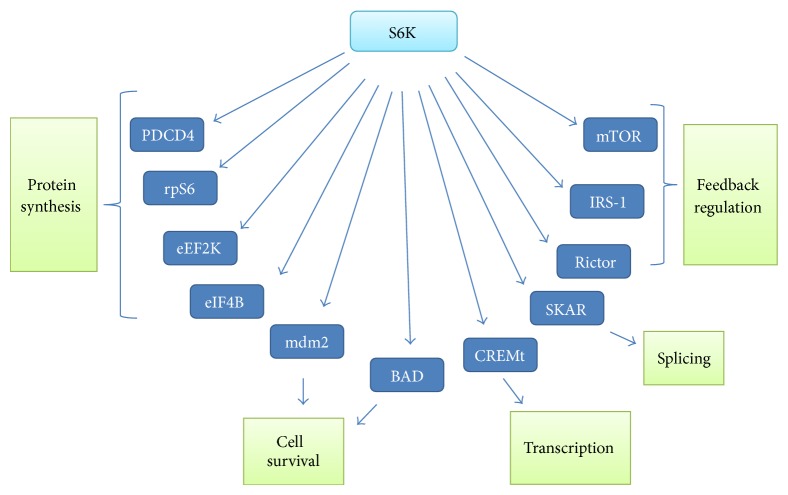
Major substrates and functions of ribosomal protein S6K.

S6K1 targets a number of proteins that control protein translation (Figure 3). S6K1 regulates translation initiation by phosphorylating the cap binding complex component eIF4B at S422 [70]. It also controls initiation of translation by phosphorylating PDCD4, a tumor suppressor that is a negative regulator of eIF4A [71], and targets it for degradation by the ubiquitin ligase, *β*TRCP [72]. S6K1 inactivates eukaryotic elongation factor-2 kinase (eEF2K) which is a negative regulator of eukaryotic elongation factor 2 (eEF2), by phosphorylating it at S366, and thus regulates the elongation step of translation [73]. Eukaryotic translation initiation factor 4B (eIF4B) is also phosphorylated by S6K1 and this phosphorylation promotes the recruitment of eIF4B to eukaryotic initiation factor 4A (eIF4A) at the translation initiation complex where it functions as a cofactor of eIF4A and increases its processivity [74]. S6K1 controls transcription by phosphorylating the cAMP response element binding protein (CREB) isoform, CREMt, and also regulates ribosome biogenesis by phosphorylating the transcription factor UBF-1 which results in the activation of RNA pol1 mediated transcription of genes that encode rRNA [75, 76]. S6K1 directly phosphorylates estrogen receptor (ER*α*) to stimulate its transcriptional activity in breast cancer cell lines [77]. S6K1 also phosphorylates the p53 ubiquitin ligase, Mdm2, on S166 [78] and proapoptotic protein BAD, thus regulating cell survival [79]. S6K1 regulates mRNA processing since it phosphorylates SKAR (S6K1 Aly/REF-like target) which is involved in mRNA splicing [80].

### 3.2. 4E-BP1

4E-BP1, which belongs to a family of three small (10–12 kda) proteins that act as inhibitors of translation initiation by binding and inactivating eIF4E, is the second well-characterized mTORC1 target. In eukaryotic cells, the mRNAs transcribed by RNA pol II have a cap structure (m7Gppp) at their 5′ end. This cap structure has several functions such as pre-mRNA processing, mRNA stability, and its export and translation. As the mRNA is exported into the cytoplasm, its cap structure interacts with the eIF4F complex, whose main function is to facilitate the recruitment of ribosome to the 5′ end of mRNA and the consequent initiation of translation. The eIF4 complex is composed of three polypeptides: eIF4E (the cap binding protein), eIF4A (an RNA helicase), and eIF4G (scaffolding protein). eIF4G interacts simultaneously with both eIF4E and eIF4A along with eIF3 which is a multiprotein complex that is associated with the 43S ribosomal particle. Briefly, the eIF4F complex facilitates the association of mRNA with ribosome by interacting with mRNA 5′ cap via eIF4E, unwinds the mRNA secondary structures via eIF4A, and recruits the ribosome through eIF4G-eIF3 interaction, thus acting as a cap dependent translation initiation factor [81].

eIF4E is regulated by phosphorylation as well as its sequestration by eIF4E binding proteins (4E-BPs). These eIF4E inhibitory proteins as well as eIF4G possess a conserved amino acid motif (YxxxxLΦ) which is the eIF4E binding motif [82]. Thus 4E-BPs compete with eIF4G for binding to the same site on eIF4E, thereby preventing the assembly of eIF4F complex and inhibiting initiation of cap dependent translation [83]. eIF4E is phosphorylated at S209 by mitogen activated protein kinase (MAPK) interacting protein kinase Mnk1/2 which uses a docking site in the carboxy-terminus of eIF4G to phosphorylate eIF4E, thus making it certain that eIF4E is phosphorylated only after the assembly of the eIF4F complex [84].

**Figure 4:**
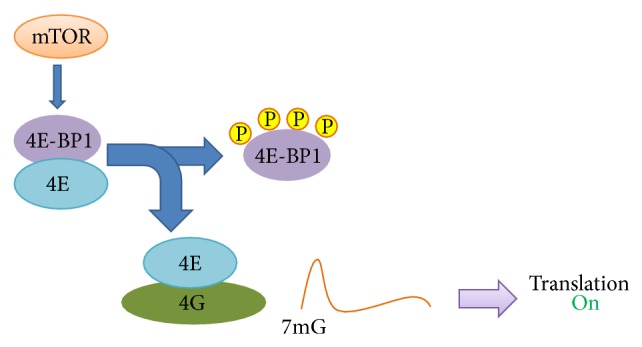
Regulation of cap dependent translation.

mTORC1 phosphorylates 4E-BP1 at several residues which promotes the dissociation of eIF4E from 4E-BP1 consequently mitigating the inhibitory effect of 4E-BP1 on eIF4E dependent translation initiation whereas the inhibition of mTOR by rapamycin is believed to cause 4E-BP1 dephosphorylation, which results in inhibition of protein translation (Figure 4) [85]. The main phosphorylation sites that have been identified in 4E-BP1 are T37, T46, S65, T70, S83, S101, and S112 [86] but the ability of 4E-BP1 to bind and inhibit eIF4E is mainly regulated by the phosphorylation of four residues: T37, T46, S65, and T70. In HEK293 cells, T37 and T46 were earlier shown to be phosphorylated significantly even in the absence of serum and the phosphorylation at these residues increased slightly with serum stimulation [87]. The phosphorylation of T37 and T46 was reported to be required for the phosphorylation of some unknown serum sensitive sites since the mutation of T37 and T46 to alanine residues prevented the phosphorylation of these serum sensitive sites while their substitution by glutamic acid residues restored the phosphorylation of the same serum sensitive sites to some extent [87]. Subsequently S65 and T70 were identified as the serum sensitive phosphorylation sites of 4E-BP1 and the phosphorylation of 4E-BP1 in HEK 293 was reported to occur in a hierarchical order. The phosphorylation of T36/T47 acts as the priming step which is followed by the phosphorylation of T70 and finally S65 [5]. However during ischemic stress in brain tissues, a new hierarchical phosphorylation for 4E-BP1 has been proposed in which T70 phosphorylation is the priming event for subsequent phosphorylation of T36/T47 [88].

S101 is necessary for phosphorylation of S65 and S112 has been shown to affect binding to eIF4E although it does not affect phosphorylation at other sites. The kinase responsible for the phosphorylation of these two serine sites has not been identified. S83 is conserved in all three 4E-BPs but does not seem to control translation initiation. The exact function and regulation of each phosphorylation site and which phosphorylations are dependent on one another are still not very well understood [89].

**Figure 5:**
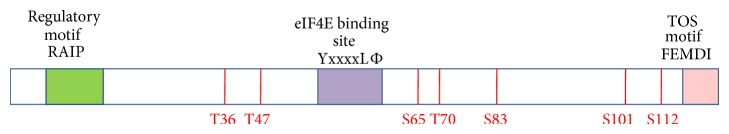
Important domains and phosphorylation sites of 4E-BP1.

Besides the eIF4E binding motif, there are two other key regulatory motifs in 4E-BP1, a RAIP motif, named after its sequence Arg-Ala-Ile-Pro at the N-terminus, and an mTOR signaling (TOS) motif at the C-terminus (Figure 5) [90, 91]. The TOS motif is also present in S6K1 and PRAS40, the other substrates of mTORC1 [27], and this motif is required for the interaction of 4E-BP1 with raptor since the mutation of specific residues within this motif abrogates the binding of raptor to 4E-BP1 [91, 92]. The RAIP motif has been found to be unable to bind with raptor [91] although another study reported that raptor does not bind to a 4E-BP variant with disrupted RAIP motif [93]. This motif is necessary for phosphorylation of residues present in both the N-terminus and the C-terminus of 4E-BP1. On the other hand, the TOS motif primarily affects the phosphorylation of S65 and T70, while the phosphorylation of the N-terminal T36/T47 residues is not affected by inactivation of the TOS motif to a great extent [91]. Also the phosphorylation of these sites is rather insensitive to rapamycin which could suggest that these phosphorylations are possibly mTOR independent however since these phosphorylations are inhibited by amino acids starvation of cells and by mTOR kinase inhibitors like wortmannin, activated by Rheb (an mTOR activator), and are decreased in mTOR knockdown cells which suggests that the phosphorylation of these N-terminal residues is mediated by mTOR but through a raptor independent mechanism [94]. Recently it has been shown that, in addition to the YxxxxLΦ motif, other conserved regions in the N- and C-termini of 4E-BP1 might also be involved in its binding to eIF4E [95, 96].

Although mTORC1 has been implicated in the regulation of 4E-BP1 phosphorylation there are various conflicting reports. For instance, rapamycin treatment leads to loss of phosphorylation at S65 and T70 on 4E-BP1 while mTORC1 has a modest effect on the phosphorylation of these sites* in vitro* [12]. On the other hand T37 and T46 are phosphorylated* in vitro* by mTORC1 but these sites are considered rapamycin insensitive in cells [12]. It is possible that rapamycin does not inhibit mTORC1 dependent phosphorylation of 4E-BP1 completely. Also it is not clear whether the rapamycin sensitive sites of 4E-BP1 are directly phosphorylated by mTOR* in vivo*; moreover some studies suggest that another unidentified 4E-BP1 kinase might exist [94]. In different cell types originating from solid or hematological tumors where 4E-BP1 phosphorylation becomes resistant to rapamycin, the Pim-2 serine/threonine kinase has been found to phosphorylate 4E-BP1 at S65 in a raptor-independent and rapamycin-insensitive way [97]. Whether this Pim-2 kinase dependent and rapamycin insensitive phosphorylation of 4E-BP1 is carried out by a rapamycin insensitive mTORC1 complex or somehow by the mTORC2 complex is not known [98].

There are some major discrepancies regarding the identity of phosphorylation sites required for the release of 4E-BP1 from eIF4E. T37 and T46 phosphorylations have been reported to have either little effect on eIF4E binding [85, 87] or to cause a major reduction in eIF4E binding affinity [99] while the effect of S65 phosphorylation on 4E-BP1 binding to eIF4E also remains in question [86, 100]. Moreover a 4E-BP1 variant that mimics hyperphosphorylation of the four main phosphorylation sites does not release eIF4E from 4E-BP1 in sea urchin, therefore suggesting that other mechanisms in addition to the phosphorylation at these four sites might play a role in 4E-BP1 binding to eIF4E [101].

Rapamycin resistant phosphorylation of 4E-BP1 has been reported in regenerating rat livers where rapamycin inhibits activation of S6K1 in response to partial hepatectomy, but 4E-BP1 phosphorylation remains uninhibited, suggesting that the effect of rapamycin on 4E-BP1 function* in vivo* can be significantly different from its effect in cultured cells [102]. Rapamycin has also been reported to differentially regulate S6K1 in comparison with 4E-BP1 in various cell lines including HeLa, MEFs, and HEK293, where rapamycin initially decreased 4E-BP1 phosphorylation but this decrease recovered within 6 h while S6K1 phosphorylation continued to be inhibited and by 12 h after treatment since 4E-BP1 was mostly hyperphosphorylated, it dissociated from eIF4E leading to a recovery in cap dependent translation even though S6K1 inhibition by rapamycin continued [103]. Several mechanisms such as association/dissociation of mTOR associated proteins or posttranslational modifications on mTORC1 have been put forward to explain this phenomenon of differential phosphorylation [104]. Torin is an ATP-competitive inhibitor that inhibits 4E-BP1 phosphorylation more strongly than rapamycin in various human cell lines. It inhibits phosphorylation of T37/T46 as well as S65 but fails to inhibit T70 phosphorylation in MEFs. These studies with Torin suggest that rapamycin resistance of mTORC1 might be a general feature of a good number of mammalian systems [105]. The activation of the tumour suppressor protein p53 induces a proteasome mediated specific cleavage of 4E-BP1 that gives rise to an N-terminally truncated, completely unphosphorylated and more stable form of 4E-BP1 that interacts with eIF4E in preference to full-length 4E-BP1, which results in long-term unavailability of eIF4E, thereby contributing to the growth-inhibitory and proapoptotic effects of p53 [106, 107]. 4E-BP1 activity is regulated in apoptosis since treatment of cells with DNA damaging drugs such as etoposide and staurosporine results in dephosphorylation of 4E-BP1 due to inhibition of mTOR signaling which impairs cap dependent protein translation and drives the IRES-mediated cap independent protein synthesis [108]. One mechanism through which DNA damage could result in the inhibition of mTOR involves p53 [109] while another different link between DNA damage and mTOR signaling is the tyrosine kinase c-Abl, which once activated by DNA damage can inactivate mTOR [110].

Various growth-inhibitory conditions and physiological stresses also lead to shutdown of mTOR signaling and 4E-BP1 dephosphorylation, which indicates that this may be a common response of cells to unfavorable conditions [111].

## 4. Other Cellular Processes Downstream of mTORC1

mTORC1 upregulates protein synthesis by various other mechanisms. Maf1 which is a Pol III repressor is inhibited by mTORC1 phosphorylation and thus induces 5S rRNA and tRNA transcription [112, 113]. The regulatory element tripartite motif containing protein-24 (TIF-1A) is activated by mTORC1, which enhances the expression of ribosomal RNA (rRNA) by promoting TIF-1A interaction with RNA Pol I [114].

Proliferating cells also require lipids in addition to protein to synthesize plasma membranes and other macromolecules and mTORC1 controls this synthesis of lipids [115] via the transcription factors SREBP1/2 (sterol regulatory element binding protein 1/2) that regulate the expression of genes involved in fatty acid biosynthesis. The inhibition of mTORC1 reduces SREBP1/2 levels which results in the downregulation of lipogenic genes [116–118]. Lipin-1 lowers SREBP1/2 levels inside the nucleus and mTORC1 mediated phosphorylation of Lipin-1 prevents it from entering the nucleus, thus suppressing this inhibition [119]. The peroxisome proliferator-activated receptor *γ* (PPAR-*γ*) is the main regulator of adipogenesis which is also activated by mTORC1 [120, 121]. mTORC1 positively regulates cellular metabolism and ATP production by activating the transcription and translation of hypoxia inducible factor 1*α* (HIF1*α*) which is a positive regulator of many glycolytic genes [116, 122].

mTORC1 is also an important negative regulator of autophagy, a eukaryotic homeostatic process in which various cytoplasmic components such as damaged organelles and intracellular pathogens are degraded inside lysosomes [123]. mTOR induces autophagy in response to reduced growth factor signalling, starvation, and other metabolic and genotoxic stresses [124] which leads to the formation of phagophores, inside which the lysosomal hydrolases degrade the targeted substrates [125]. During physiological conditions, the phagophore formation is inhibited by mTORC1, since it directly interacts with and phosphorylates the Ulk1 kinase complex (Ulk1-Atg13-FIP200-Atg101) which is required for the initiation of autophagy [126, 127]. mTORC1 regulates WIPI2 (mammalian orthologue of Atg18), which is also essential for phagophore formation [128], as well as DAP1 (death associated protein 1), an inhibitor of autophagy [129]. During unfavourable conditions, such as cell starvation or rapamycin mediated inhibition of mTOR kinase, the Ulk1 complex is released from mTOR, thereby allowing it to associate with the membranes from which phagophores are formed [130].

## 5. The mTOR Pathway and Cancer

The mTOR pathway is related to tumorigenesis because of its vital role in cell growth, proliferation, and metabolism. The aberrant activation of mTOR pathway either by loss of tumor suppressors or activation of oncogenes promotes tumor growth in various malignant cell lines. The upstream and downstream elements of the mTOR pathway are dysregulated in different human cancers. The overexpression of different growth factor receptors like IGFR (insulin like growth factor receptor) and HER2 (human epidermal growth factor receptor 2) and mutations in the PI3K can lead to activation of AKT and mTOR pathways [131]. This mTOR activation causes an increase in ribosome biogenesis that promotes cell proliferation by providing the machinery which is required by cells to maintain high levels of growth [132].

The downstream effectors of mTORC1, 4E-BP1, eIF4E, and S6K1 are also associated with various malignancies. eIF4E is an oncogene as it is overexpressed in many human cancers with poor prognosis and its overexpression results in transformation of cells* in vivo* [133, 134]. eIF4E promotes the translation of specific mRNAs that code for prooncogenic proteins which promote cell survival and cell-cycle progression, energy metabolism, and metastasis, thus affecting cell proliferation and tumorigenesis [135]. Dysregulated expression as well as increased phosphorylation of 4E-BPs in cancer also results in poor patient prognosis and the loss of 4E-BP1 with the resulting activation of cap dependent translation promotes cell-cycle progression and cell proliferation in culture [136, 137] whereas overexpression of constitutively active 4E-BP1 suppresses tumor growth* in vivo* [138, 139]. The increase in cell proliferation by downregulation of 4E-BP1 might be due to the removal of the inhibition of translation of mRNAs that encode proteins such as vimentin, Y-box protein, and CD44, which promote cell growth, proliferation, and metastasis [140]. S6K1 is also overexpressed in lung and ovary cancer [141] and its gene expression has been found to be upregulated in brain tumors [142].

mTORC2 also plays a role in cancer since the mTORC2 subunit RICTOR is also overexpressed in multiple cancer types [143] and its overexpression increases mTORC2 activity which causes the cancer cells to become more proliferative and invasive [143, 144]. Dysregulation of several other elements of the mTOR pathway also results in tumorigenesis. The loss of the tumor suppressor PTEN decreases its expression in many human cancers [145] and in mice results in the development of prostate cancer [146]. PROTOR 1 is downregulated in human breast tumors and cell lines [147] whereas deptor is overexpressed in various tumors such as myelomas and hepatocellular carcinomas [43, 148] and RHEB is overexpressed in some human lymphomas [149, 150].

Lipid synthesis is increased in proliferating cancer cells and PI3K/AKT mediated high glycolytic rates produce ATP and the other building blocks required for lipid synthesis [151]. The mTORC2 substrate GSK3 (glycogen synthase kinase 3) connects AKT to lipid synthesis since GSK phosphorylates lipogenic transcription factor SREBP (sterol responsive element binding protein) and targets it for protein degradation which is opposed by AKT mediated phosphorylation and inactivation of GSK. Thus dysregulated AKT/mTOR pathway can promote SREBP expression and activity which in turn enhances cancer cell lipid biosynthesis [116].

Activated mTOR pathway is also related to various familial cancer syndromes. The loss of tumor suppressor LKB1 (liver kinase B1), which is a key kinase for activating AMPK [152], results in Pentz-Jeghers syndrome [153]. Mutations in TSC1 or TSC2 cause tuberosis sclerosis [154], another familial cancer syndrome, and PTEN mutations result in Cowden's syndrome [155]. These syndromes result in benign tumors which may progress to malignancies.

## 6. Targeting the mTOR Pathway in Cancer

mTOR inhibitors can be broadly grouped into two classes: the allosteric inhibitors of mTORC1 (rapamycin and rapalogs) and the mTOR kinase inhibitors.

### 6.1. Rapamycin and Rapalogs

Rapamycin was the first mTOR inhibitor but despite its antitumor activity in preclinical models it was not successful as an anticancer drug. Subsequently several analogs of rapamycin, now called rapalogs, with better solubility and pharmacokinetic properties were synthesized such as temsirolimus, everolimus, and deforolimus. Like rapamycin these rapalogs form a complex with intracellular receptor FKBP12 which binds to mTOR and inhibits mTORC1 downstream signaling. In phase II and III clinical trials, everolimus and temsirolimus were effective in treating RCC (renal cell carcinoma), neuroendocrine tumors, and Mantle cell lymphoma and both have been approved by FDA for treatment of RCC [156]. However, the success of rapalogs as anticancer monotherapies was limited because the FKBP12-rapamycin complex cannot bind to mTORC2 [41] and also due to the activation of alternative signaling pathways such as the MAPK pathway [157] and AKT signaling resulting from the loss of a negative-feedback mechanism. Another reason why rapamycin and its analogs have limited efficacy in cancer treatment is because these drugs only partially inhibit the phosphorylation of 4E-BP1 [105, 158–160]. In AML, for example, the phosphorylation of S6K1 on T389 is abrogated by RAD001 while phosphorylation of 4E-BP1 on S65 residue remains unaffected, thus failing to inhibit mRNA translation and the assembly of eIF4F complexes [97].

### 6.2. Catalytic mTOR Inhibitors

In order to overcome the limitations of rapalogs, ATP-competitive mTOR kinase inhibitors were developed that directly target the mTOR catalytic site and inhibit phosphorylation of mTORC1 substrates S6K1 and 4E-BP1 as well as phosphorylation of the mTORC2 substrate AKT and mTORC2 [161]. These inhibitors include Ku-006379, Torin, PP242, PP40, OSI027, AZD2014, and AZD8055 which show better antitumorigenic effects in comparison with rapalogs because they bind to the ATP binding site of mTOR, thus inhibiting the catalytic activity of mTORC1 and mTORC2 [105, 159, 162]. The anticancer activity of these inhibitors has been superior to rapamycin in preclinical trials due to effective blocking of cell proliferation, 4E-BP1 phosphorylation, and protein translation [159]. Two such inhibitors, PP242 and PP40 which inhibit the insulin stimulated S473 phosphorylation of AKT, also inhibit protein synthesis and cell proliferation [159]. PP242 and OSI-027 show superior anticancer effects in BCR-ABL expressing cell lines [163]. OSI-027 is more potent in blocking 4E-BP1 phosphorylation and mRNA translation in acute myeloid leukemia in comparison with rapamycin [164]. Torin 1 is a selective ATP competitive inhibitor that is also more effective in inhibiting cell growth and proliferation than rapamycin [105]. AZD8055 potently (IC < 1 nM) inhibits the rapamycin-resistant T37/46 phosphorylation sites on 4E-BP1, resulting in significant inhibition of cap dependent translation.* In vitro*, AZD8055 effectively inhibits cell proliferation and induces autophagy and,* in vivo*, AZD8055 inhibits tumor growth [158]. In addition, it combines well with the MEK1/2 inhibitor selumetinib in preclinical studies [165]. Significant phosphorylation of 4E-BP1 at T37/T46 and S65 is still observed in mTORC2 deficient Sin-1 knockout MEFs when treated with rapamycin, even though the hydrophobic motifs of both AKT and S6K1 are not phosphorylated due to absence of mTOR activity in both of its complexes. In contrast, exposure of Sin-1 knockout MEFs to Ku-0063794 dephosphorylates 4E-BP1 to a much greater extent than rapamycin [160]. Treatment of cells with Torin and PP242 inhibitors indicates that antiproliferative effects of these mTOR kinase inhibitors are primarily through disruption of mTORC1 functions which are resistant to rapamycin [118].

The drawback with mTOR kinase inhibitors as with rapalogs is that the mTORC1 feedback loop can be relieved which leads to activation of PI3K or MAP kinase signaling [166]. Thus dual specificity drugs that target both mTOR function and AKT activation can improve antitumor activity. Also rapalogs as well as catalytic inhibitors can lead to induction of autophagy which can be anti- as well as protumorigenic depending upon stimulus [167]. Autophagy has been recently shown to enable survival to mTOR inhibition [168]. Therefore combination treatment of cancers with rapalogs or catalytic inhibitors along with autophagy inhibitor may be a better strategy [18].

Another mechanism that explains the resistance to mTOR kinase inhibitors is that cancer cells downregulate the expression of 4E-BPs which leads to an increase in the eIF4E/4E-BP1 ratio, and it is this change in eIF4E/4E-BP1 stoichiometry that limits the sensitivity of cancer cells to catalytic site TOR inhibitors suggesting that the eIF4E/4E-BP1 ratio might act as a predictive marker for treatments using catalytic TOR inhibitors [169].

## 7. Dual PI3K/mTOR Inhibitors

Dual PI3K/mTOR inhibitors were developed because of the above concerns over mTOR inhibitors. This was made possible because of the high homology that is shared by the kinase domains of PI3K and mTOR [16]. These molecules inhibit mTORC1, mTORC2, and PI3K, thus inhibiting the phosphorylation of AKT, S6K1, and 4E-BP1, and are therefore attractive drugs for targeting cancers driven by PI3K activation [170]. These inhibitors include XL-765, PI-103, and NVP-BEZ235 which are undergoing phase I/II clinical trials [171, 172]. PI-103 and NVP-BEZ235 have been found to suppress AKT as well as S6K1 in breast tumors and leukemia cells [173], although some studies suggest that such broad inhibition of cellular signalling may also impair growth of normal cells [174].

## 8. Concluding Remarks

mTOR pathway plays a key role in nutrient homeostasis that regulates cellular growth and proliferation. mTOR regulates protein translation through effector molecules S6K1 and 4E-BP1. Dysregulation of the pathway is complicated by cross-talk between mTOR and other signalling pathways like AKT and PI3 kinase. Though mTOR pathway dysregulation manifests into various pathological states, it is not the only candidate responsible for the effect. Further downstream signalling is very complex that is understood by the fact that therapeutic regimens that target only mTOR are not very effective to treat cancer. Dual inhibitors that target both mTOR and PI3 kinase have shown promise in combating the disease.
